# Ovarian Cancer—Why Lipids Matter

**DOI:** 10.3390/cancers11121870

**Published:** 2019-11-26

**Authors:** Guangyuan Zhao, Horacio Cardenas, Daniela Matei

**Affiliations:** 1 Department of Obstetrics and Gynecology, Feinberg School of Medicine, Northwestern University, Chicago, IL 60611, USA, Guangyuan.Zhao@northwestern.edu (G.Z.); horacio.cardenas@northwestern.edu (H.C.); 2 Robert H. Lurie Comprehensive Cancer Center, Feinberg School of Medicine, Northwestern University, Chicago, IL 60611, USA; 3 Hematology Oncology, Jesse Brown VA Medical Center, Chicago, IL 60612, USA

**Keywords:** ovarian cancer, lipid metabolism, stem cell

## Abstract

This review highlights recent advances in the understanding of the relevance of altered lipid metabolic pathways contributing to the poor prognosis of high grade serous ovarian cancer, as they relate to cancer metastasis and cancer stemness. Increased lipid uptake regulated by the receptor CD36 and the transport protein FABP4 has been implicated in ovarian cancer metastasis. The symbiotic relationship between ovarian cancer cells and adipocytes was shown to be important for sustaining widespread peritoneal and omental metastasis. Increased lipogenesis dependent on the fatty acid desaturase SCD1 was detected in ovarian cancer stem cells. Furthermore, response to therapy, specifically to platinum, was linked to increased fatty acid biogenesis, while the survival of drug tolerant cells was shown to depend on lipid peroxidation. These recent findings suggest that lipids are necessary elements supporting oncogenic signaling and the energetic needs of rapidly proliferating cancer cells. New strategies targeting key enzymes involved in lipid uptake or utilization in cancer cells have been shown to exert anti-tumor effects and are being developed as cancer interventions in combination with chemotherapy or immunotherapy.

## 1. Introduction

Epithelial ovarian cancer (OC), an aggressive tumor with origins in the fallopian tube epithelium [1], is characterized by the propensity of metastasizing early and presenting with disseminated implants in the peritoneal cavity and infiltrating the omentum, a fat rich organ. Due to the widely metastatic presentation, OC is rarely curable and most patients succumb to the disease. The most common histological subtype is high grade serous ovarian cancer (HGSOC), which is characterized by a p53 mutated signature and deficiency in homologous recombination [2]. Because of the DNA repair mechanisms commonly deficient in HGSOC, these tumors are initially very chemo-responsive to platinum-based therapy [3,4]. Treatment personalization that takes into account clinical characteristics such as performance status, age, and histological type of tumors [5,6], as well as molecular characteristics [7] is evolving. Most patients reach meaningful near-complete responses and sustained remissions; however, eventually the majority of women with OC relapse and recurring tumors become chemo-resistant and ultimately fatal [4,8].

Many mechanisms have been implicated in development of acquired platinum resistance, including export pathways, epigenetic modifications, and alterations in DNA damage response. More recently, it has been hypothesized that a key phenomenon implicated in disease recurrence in OC and other solid tumors is the persistence of cancer stem cells (CSCs), which are quiescent and therefore can escape the effects of cytotoxic therapy, survive and under the stimulation of certain factors in the peritoneal environment, eventually become reactivated and give rise to recurrent tumors, which are heterogeneous and highly treatment-resistant [9,10,11]. Understanding the unique characteristics of CSCs has been deemed a priority [12].

Metabolic alterations have been associated with rapid growth of tumors, early propensity to metastasize, development of resistance to therapy, and survival of CSCs. While abnormal glycolysis and glucose metabolism are well understood in these contexts, new data related to the role of lipid metabolism in cancer are emerging. Due to the almost symbiotic relationship between OC and the fat containing cells in the omentum, alterations of the lipid metabolism in HGSOC remain of high interest both to advance the understanding of the mechanisms that fuel peritoneal dissemination, and to identify potential new targets for therapeutic interventions. Here, we review recent advances in the field related to lipid metabolic pathways altered in cancer of specific relevance to HGSOC.

## 2. Cellular Lipid Metabolism

**Figure 1:**
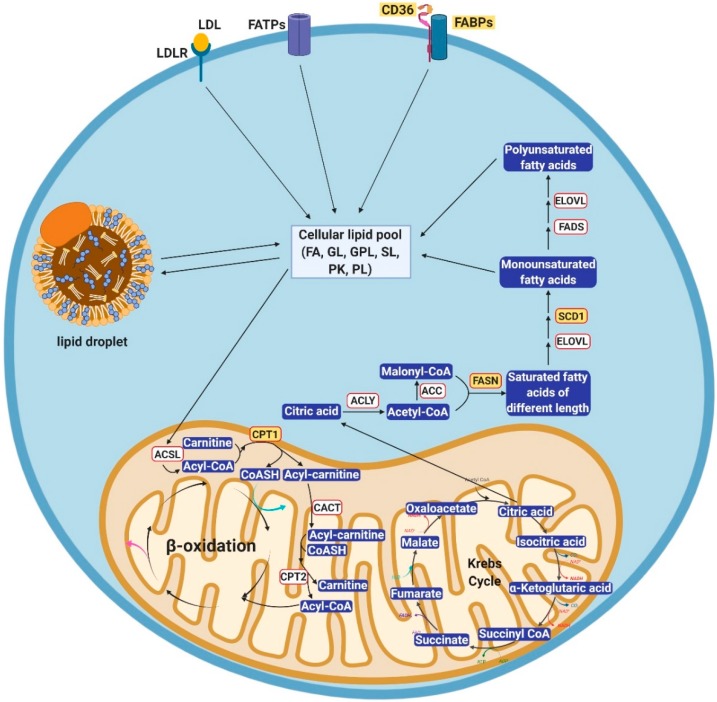
Cellular fatty acid uptake, anabolism and catabolism. Overview of the metabolic pathways involved in fatty acid uptake, de novo lipogenesis, and β-oxidation. In the top part, the known uptake routes for fatty acids, contributing to the cellular free fatty acid pool are shown and include the low-density lipoprotein receptor, fatty acid transporter proteins, fatty acid translocase, and fatty acid binding proteins. In the center part, the conversion between the free fatty acid pool and lipid droplets is depicted. In the bottom part, oxidation and biosynthesis of fatty acids in the mitochondria are illustrated. The magenta and cyan arrows in the β-oxidation cycle indicate generation of FADH_2_ and NADH. Citric acid generated from the Krebs Cycle diffuses into the cytoplasm to participate in de novo lipogenesis. Highlighted proteins are those found to be significant in ovarian cancer. Lipid species: FA, fatty acyls; GL, glycerolipids; GPL, glycerophospholipids; SL, sphingolipids; PK, polyketides; PL, prenol lipids. Enzymes: ACC, acetyl-CoA carboxylase; ACLY, ATP-citrate lyase; ASCL, ATP-dependent acyl-CoA synthetase; CACT, carnitine-acylcarnitine translocase; CD36, fatty acid translocase; CPT1, carnitine palmitoyl transferase 1; CPT2, carnitine palmitoyl transferase 2; ELOVL, fatty acid elongase; FADS, fatty acid desaturase; FASN, fatty acid synthase; FABPs, fatty acid binding proteins; FATPs, fatty acid transport proteins; LDL, low-density lipoprotein; LDLR, low-density lipoprotein receptor; SCD1, stearoyl CoA desaturase 1. Image was created by using BioRender (https://biorender.com/).

Lipids are hydrophobic biomolecules which include fatty acyls, glycerolipids, glycerophospholipids, sphingolipids, saccharolipids, polyketides, sterol lipids, and prenol lipids [13]. Three major routes play a role in how lipids are routed and used inside the cell: uptake, lipogenesis, and utilization (Figure 1). The metabolism of lipids is closely aligned with that of glucose and tightly regulated by enzymes which are rate limiting at various steps.

Lipids are imported into cells through a variety of fatty acids transporters present on the plasma membrane [14,15]. These transporters include low-density lipoprotein receptors, fatty acid transport proteins (FATPs), fatty acid translocase, and fatty acid binding proteins (FABPs). Low-density lipoprotein receptors bind to low-density lipoproteins in blood and transport them inside the cells, where cholesterol is released after the low-density lipoproteins are broken down [16]. The fatty acid transport protein family includes six members (FATP1-6) with distinct distribution across tissue types [14]. FATPs contain a functional AMP-binding motif [17,18] which actively participates in fatty acid transport. Fatty acid translocase, also known as CD36, is the predominant fatty acid transporter in many normal cell types, including adipocytes, cardiac myocytes, enterocytes, and skeletal myocytes (reviewed in [19]) and also in cancer cells. Most of the time, CD36 works in concert with FABPs to facilitate the import of fatty acids. FABPs are fatty acid chaperones, which possess distinct patterns of expression [15]. Thus far, nine FABP family members have been identified, including adipocyte, brain, epidermal, heart, intestinal, ileal, liver, myelin, and testis FABPs. The plasma membrane-bound domain of FABPs interacts with the transmembrane domain of CD36 to facilitate the import of free fatty acids from the extracellular space. Once inside the cell, fatty acids remain bound to the cytosolic part of FABPs until their delivery to the destination site for storage in lipid droplets, oxidation in mitochondria, lipid synthesis in the membrane, or use as transcriptional regulators in the nucleus.

Lipogenesis is the pathway by which lipids are generated in the cell from other metabolites. This pathway is typically activated when exogenous lipids are unavailable for import, such as during starvation or energy shortage. In these situations, the cell uses alternative metabolic units to build fatty acids. For example, cytosolic citrate resulting from glutamine metabolism and citric acid cycle downstream of glucose metabolism is cleaved by ATP-citrate lyase into acetyl-CoA [20]. Acetyl-CoA carboxylase (ACC) then turns this metabolite into malonyl-CoA which, together with acetyl-CoA, is condensed into saturated fatty acids of various lengths by the fatty acid synthase (FASN) [21]. These initially generated fatty acids undergo further elongation mediated by the fatty acid elongases [22]. These newly generated fatty acids are saturated, bearing single carbon-carbon bonds. Stearoyl CoA desaturases (SCDs), SCD1 and SCD5 then convert palmitic acid and stearic acid, two of the major free fatty acids generated by FASN and fatty acid elongases into palmitoleic and oleic acid, respectively, creating double carbon-carbon bonds at Δ9 position [23,24]. Palmitoleic and oleic acids are further reduced into polyunsaturated fatty acids by other fatty acid desaturases. The majority of those fatty acid species are not present in the cytoplasm in the form of free fatty acids; rather, they are esterified with glycerol into triglycerides and stored in lipid droplets [25]. De novo synthesized fatty acids participate in different aspects of cellular physiology, such as construction of cell membranes, biogenesis of energy-storing lipids, and formation of signaling molecules through post-translational fatty acylation of proteins [26].

Fatty acids represent an important source of fuel in the cell, which is released through oxidation, tightly regulated by several enzymes. Free fatty acids in the mitochondrial inter-membrane space are converted into acyl-CoA by ATP-dependent acyl-CoA synthetase. Carnitine palmitoyl transferase 1 tags fatty acyl-CoA with carnitine to form fatty acyl-carnitine which is transported into the mitochondrial inner membrane via carnitine-acylcarnitine translocase. Once inside the mitochondrial matrix, fatty acyl-CoA is reformed by carnitine palmitoyl transferase 2 with free CoA. The subsequent fatty acid β-oxidation strips one acetyl-CoA group off fatty acyl-CoA at a time, generating FADH_2_ and NADH as small and mobile energy storage units that participate in different biochemical processes including the generation of ATP via the electron transport chain [27]. Through this process, the cell utilizes the stored energy in the form of fatty acids for its metabolic needs. In cancer, these processes need to be augmented to keep up with the increased energetic requirements of growing tumors or of tumors stressed by various cancer treatments.

### 2.1. Alterations of Lipid Uptake in Cancer

Cancer cells utilize various strategies to boost lipid uptake in order to fulfil their high energetic needs for cell growth and altered oncogenic signaling. Alterations in lipid uptake were described in different cancer types, including HGSOC. For example, breast cancer cells were shown to import exogenous oleic acid through upregulated CD36 and this mechanism alleviated the apoptotic effects induced by SCD1 inhibition [28]. The very low-density lipoprotein receptor was found to be overexpressed in clear-cell renal cell carcinoma in a HIF1α-dependent manner [29], leading to lipid accumulation in tumor cells. Similar observations were reported by Bensaad et al. in a VEGF inhibitor-resistant xenograft mouse model of glioblastoma [30]. Chronic VEGF inhibitor therapy was shown to strongly induce the expression of FABP3 and FABP7, resulting in increased lipid uptake and storage of fatty acids in lipid droplets. Another report described upregulation of FABP5 in malignant prostate cancer versus normal tissue [31]. Stable knockdown of FABP5 in prostate cancer cells significantly reduced the tumor burden in a xenograft mouse model, highlighting the significance of lipid uptake to tumor growth in vivo. Pascual et al. found that in an oral carcinoma model, among slow cycling tumor initiating cells identified by CD44 expression, there was a small subpopulation with increased metastatic potential, which was characterized by abnormal lipid metabolic features. In particular, these cells were found to have high expression of CD36 and were sufficient and necessary for metastasis in oral carcinoma [32]. CD36^+^ oral carcinoma cells could initiate tumor metastasis more effectively than CD36^−^ cells, while retaining the same ability to generate tumors at the primary site. Interestingly, tumor metastasis was augmented by a fat rich diet, in a CD36-dependent manner. Strategies targeting CD36 by using neutralizing antibodies decreased the metastatic potential [32], pointing to a potential metabolic vulnerability of these highly tumorigenic cells.

The role of lipid uptake has also been studied in ovarian cancer. Recognizing that the preferred site of metastasis in ovarian cancer is the omentum, a fat rich organ, Lengyel’s group characterized the symbiotic relationship between adipocytes and ovarian cancer cells [33]. Direct transfer of lipids between adipocytes and OC cells was shown to be mediated by FABP4, which was highly upregulated in metastatic versus primary tumor sites. Depletion of FABP4 was sufficient for diminishing the metastatic potential of HGSOC cells. More recently, FABP4 was described by others as being critical to mediating the metastatic potential of ovarian cancer both in in vitro cell migration and invasion assays and in in vivo orthotopic mouse models [34]. Bioinformatics analyses of the TCGA dataset using chemotherapy-naïve cases of HGSOC demonstrated that the expression level of the microRNA miR-409-3p was negatively correlated with that of FABP4. Mir-409-3p was further predicted to bind to the 3′UTR of FABP4 and was validated as a key regulator of FABP4. Metabolomics experiments found higher unsaturation and oxidation of fatty acid species in high FABP4-expressing human tumor specimens, and these metabolic abnormalities were correlated with poor overall and progression-free survival. Lastly, the authors used a known inhibitor of FABP4, tamoxifen, and demonstrated that physiological concentrations of this drug impaired the uptake of free fatty acids and inhibited OC cell migration and invasion. These experimental observations correlate with clinical reports indicating that this anti-estrogen has modest anti-tumor activity in OC [34].

A more recent report focusing on lipid uptake demonstrated the significance of the CD36 transporter mediated fatty acid transport into ovarian cancer cells [35]. The authors found that human primary adipocytes induced CD36 mRNA level and plasma membrane expression in co-cultured ovarian cancer cells. Consequently, fatty acid uptake and lipid droplet accumulation were enhanced in cancer cells. Interestingly, genes involved in endogenous lipid metabolism and cholesterol biosynthesis were downregulated in tumor cells. These observations suggested that ovarian cancer cells, in the presence of primary adipocytes, rely more on the uptake of exogenous lipids and cholesterol than on de novo lipogenesis. More importantly, knockdown of CD36 suppressed baseline and adipocyte-induced cellular invasion and migration, as well as adhesion to major extracellular matrix components of the peritoneum, such as type I collagen and laminin. In vivo experiments demonstrated that CD36 knockdown or treatment with a neutralizing antibody significantly reduced tumor burden and metastatic nodules in intraperitoneal OC xenograft models. Together, these data strongly support the significance of increased lipid uptake to tumor metastasis in this cancer type.

### 2.2. Alterations of Lipogenesis in Cancer

While the excessive anaerobic metabolism of glucose, i.e., the Warburg effect [36,37] in cancer cells has been well described, as a major mean to provide energy for proliferation and survival [38], less is known in terms of lipid synthesis in cancer. Recent cumulative results have pointed to the concept that cancer cells rely on lipogenesis to adapt to cytotoxic stress in the tumor microenvironment. This is particularly important in tumor areas where the exogenous supply of fatty acids is scarce, such as in hypovascular and hypoxic regions [39,40]. Such regions are abundant in large, rapidly growing ovarian tumors. FASN-mediated de novo lipogenesis was reported in breast [41] and pancreatic cancer [42]. Breast cancer cells were shown to upregulate FASN to induce non-homologous end joining DNA repair which in turn counter-acted the genotoxic stress induced by chemotherapy and radiotherapy.

Additionally, fatty acid desaturation, a critical step in the process of lipogenesis, is essential for the maintenance of membrane fluidity, inter- and intra-cellular signaling, and provision of lipids for energy generation through oxidation [43]. Notably, SCD1 is the rate-limiting enzyme converting saturated fatty acids to unsaturated fatty acids. SCD1 was found to be upregulated in various neoplasms [44,45] and its inhibition prevented cancer cell proliferation when exogenous fatty acids were depleted [46]. Depletion of SCD1 in vivo led to reduction in hepatic lipogenesis, increased insulin sensitivity and protection from carbohydrate-induced obesity [47,48]. Beyond SCD1 mediated desaturation, the Δ6 and Δ5 desaturases in concert with elongases are involved in the synthesis of polyunsaturated fatty acids from exogenously acquired alpha-linolenic and linoleic acid [49], which are present in the inflammatory environment associated with tumor initiation.

Recently, our group developed a new imaging approach based on Raman spectroscopy that allowed us to probe the different lipid species in rare cell populations, such as stem cells [50,51]. By using this technology, we demonstrated that unsaturated fatty acids (both monounsaturated fatty acids and polyunsaturated fatty acids) were enriched in ovarian cancer stem cells [52]. We found that unsaturated fatty acids were essential for the proliferation and survival of ovarian cancer stem cells and that pharmacological inhibition of SCD1 activity or shRNA-mediated knockdown of SCD1 eliminated ovarian cancer stem cells and retarded tumor initiation. The concept of increased lipid unsaturation and the role of fatty acid desaturases in cancer progression are also supported by work from other groups in different cancer models. Overexpression of acetyl-CoA synthetase and SCD1 led to increased cellular monounsaturated fatty acids and conferred bioenergetic advantage necessary to initiate and carry out epithelial-to-mesenchymal transition (EMT). Expression levels of these enzymes were shown to be correlated with clinical outcomes in colon cancer, triple negative breast cancer, and aggressive prostate cancer [53]. SCD1 inhibitors blocked prostate and breast cancer cell proliferation in low serum conditions, when the exogenous supply of fatty acids was limited [54,55] and these effects were rescued by addition of exogenous oleic acid. Similarly, depletion of oleic acid, but not of palmitic acid, inhibited the proliferation of acute myelogenous leukemia and lymphoma cells [56].

Our current understanding of fatty acid desaturation supports that SCD1 is the sole enzyme responsible for converting saturated fatty acid to monounsaturated fatty acid. However, very recently, Vriens et al. discovered an alternative fatty acid desaturation pathway, which is operative in multiple cancer types, including lung, breast, prostate, and liver cancer [57]. In those types of cancer cells, palmitic acid was converted to sapienic acid by fatty acid desaturase 2. Additionally, inhibition of SCD1 activity accounted for less than 50% of the cancer cell proliferation. These recent findings unveiled an unknown property of cancer cells synthesizing lipids and indicate plasticity and adaptation in cancer cells. Future endeavors could be directed to identify and characterize the population of cancer cells that utilize this alternative lipid metabolism pathway to survive during SCD1 inhibition.

Furthermore, while lipid unsaturation is critical to the survival of cancer stem cells, it has also been linked to normal stem cell physiology. FASN was found to be upregulated in neural progenitors, where suppression of its activity affected normal neurogenesis [58]. Embryonic stem cells, characterized by the presence of a highly unsaturated lipidome, became differentiated in response to in vivo oxidative processes such as inflammation [59]. These findings were also corroborated by the live Raman scattering microscopy based observations showing that lipid droplets are enriched in mouse oocytes and early embryos and persist until the blastocyst stage [60]. Taken together, results from cancer biology and developmental biology support the concept that lipid unsaturation is associated with stemness.

## 3. Important Pathological Effects of Altered Lipid Metabolism in Ovarian Cancer

Abnormalities in lipid metabolism have been linked to several cancer phenotypes, including metastatic potential, cancer stemness and resistance to chemotherapy. Specific alterations and the cellular mechanisms engaged in these contexts are reviewed bellow.

### 3.1. Involvement of Lipid Metabolism in the Process of OC Metastasis

Advanced stage ovarian cancer is typically characterized by omental or peritoneal metastasis, which leads to the typical complications of the disease, ascites and bowel obstruction. The omentum is mainly comprised of adipocytes which serve as a chemical attractant for ovarian cancer cells. Nieman et al. have identified that the interleukins, IL-6, IL-8, monocyte chemoattractant protein-1, and tissue inhibitor of metalloproteinases-1 are released by the omentum to promote dissemination of ovarian cancer cells [33]. Specifically, they found that IL-8 secreted by cells from the omentum binds to CXCR1 on ovarian cancer cells to induce p38-mitogen-activated protein kinase and STAT3 phosphorylation, hence promoting initiation of metastasis. The molecular mechanism that distinguished metastatic tumors from primary tumors relied predominantly on the fatty acid binding protein 4 (FABP4). FABP4 was highly expressed on the membrane of disseminated ovarian cancer cells at the adipocyte–cancer cell interface and mediated lipid accumulation in ovarian cancer cells. Inhibition of FABP4 led to reduced intracellular lipid accumulation and adipocyte-mediated invasion capability of ovarian cancer cells. It was proposed that the accumulated lipid droplets transferred between adipocytes and cancer cells could be used through oxidation to generate ATP for energy consumption during colonization and formation of micrometastases. In addition to in vitro experiments, knock out of FABP4 in ovarian cancer cells caused decreased tumor burden and number of metastatic nodules in intraperitoneal OC mouse models, convincingly establishing the link between lipid metabolism and the metastatic phenotype.

Aside from metastatic ovarian cancer, altered lipid metabolism was also observed as a regulator of tumor metastasis to lymph nodes in melanoma by Lee et al. [61]. The authors utilized subcutaneous xenograft models and compared cells isolated from the primary tumor site versus micrometastatic and macrometastatic tumor-draining lymph nodes. RNA-sequencing analysis revealed that top up-regulated gene sets in the micro- and macro-metastatic tumors were positively correlated with bile acid metabolism, adipogenesis, fatty acid metabolism, cholesterol homeostasis, and oxidative phosphorylation. Pathway analysis demonstrated that lymph node metastatic tumors induced fatty acid oxidation and peroxisome proliferator-activated receptor-α (PPARα) signaling. Further metabolomic experiments showed that tumors metastatic to lymph nodes accumulated more fatty acid species compared to tumors at primary sites. Treatment with etomoxir, an inhibitor of fatty acid oxidation, did not affect the size of primary tumors, but it significantly suppressed lymph node metastasis. Notably, etomoxir also suppressed lymph node metastasis after removal of the primary tumors. To identify the underlying mechanism implicated in the fatty acid oxidation-dependent process of metastasis, Lee et al. knocked down each of the oncogenic genes in the metastasis-prone cells and found that knockdown of YAP1 significantly reduced fatty acid oxidation in these cells. Conversely, overexpression of YAP1 enhanced fatty acid oxidation in metastatic cells in vitro and in vivo. Immunofluorescent imaging analysis revealed that active YAP1 was localized in the nucleus of cells at the invasive front of tumors, while it was inactive, located in the cytoplasm of cancer cells, at the primary tumor site [61]. Collectively, these data convincingly support the link between lipid metabolism and metastasis in OC and other cancer types.

### 3.2. Lipid Metabolism in Ovarian Cancer Stem Cells

Cancer stem cells represent a small population residing within heterogeneous tumors and have been reported to mediate ovarian cancer initiation [62,63,64]. These cells, which represent about 1% of the tumor mass, have also been implicated in tumor progression [63], resistance to chemotherapy or radiotherapy [63,65,66], and metastasis to distant tissues [67]. The underlying signaling pathways that maintain cancer cell stemness have been studied in depth, and include the WNT-β-catenin pathway [68], the NF-κB pathway [69], and NOTCH signaling [70]. Nevertheless, less is known about the metabolic regulation of cancer stem cells due to difficulties studying these rare cells by using traditional mass spectrometry-based methods, which typically require large numbers of cells for analysis.

A recent report from our group utilized stimulated Raman Scattering Microscopy to study ovarian cancer stem cells and found that these cells harbor higher level of unsaturated fatty acids and increased levels of the SCD1 desaturase mRNA as compared to non-stem cancer cells [52]. Suppression of fatty acid desaturation by small molecule inhibitors against SCD1 downregulated stemness markers in cancer stem cells isolated from both cancer cell lines and primary patient samples. SCD1 inhibitor-treated cancer stem cells, when injected subcutaneously into athymic mice, displayed impaired in vivo tumor initiation capacity, delayed time-to-tumor formation, and reduced tumor burden. Additionally, our group found that ovarian cancer stem cells relied on de novo synthesis of unsaturated fatty acids rather than on uptake of exogenous fatty acids from the immediate environment [52].

In order to identify the molecular mechanisms linking unsaturated fatty acids and ovarian cancer cell stemness, ovarian cancer stem cells treated with vehicle and an SCD1 inhibitor were probed by using a pathway-specific quantitative RT-PCR array. Transcriptional activity of NF-κB was found to be compromised in ovarian cancer stem cells upon suppression of SCD1 desaturase activity. Furthermore, the NF-κB signaling pathway was shown to directly regulate the transcription of SCD1, through a positive feedback loop. It is still not clear whether the pathway is activated directly or indirectly by the increased amounts of unsaturated fatty acids in this context.

These observations were corroborated in other cancer types, such as colon and breast cancer [71,72] as well as glioblastoma [73]. For example, the stemness associated-transcription factor NANOG was shown to suppress mitochondrial oxidative phosphorylation and tilt the metabolic balance in tumor initiating cells towards fatty acid oxidation to support stem cell self-renewal [74]. Inhibition of lipogenesis mediated by acetyl-CoA carboxylase was shown to inhibit mammosphere generation and the ALDH+ stem cell population in a breast cancer model [75]. Elongations of very long fatty acids protein 2 (ELOVL2) an enzyme involved in the biogenesis of poly-unsaturated fatty acids was found to be epigenetically upregulated in glioma cancer stem cells. Its inhibition caused disruption of phospholipids in the plasma membrane leading to altered EGFR signaling and suppression of tumorigenicity and self- renewal in this cell population, further consolidating the concept that unsaturated fatty acids play a role in the maintenance of cancer stem cells [76]. These data suggest that targeting lipogenesis may hit the Achille’s heel in these highly tumorigenic and treatment-resistant cancer cells.

### 3.3. Altered Lipid Metabolism in the Context of Chemotherapy Resistance

Based on the significance of lipid metabolism to cancer stem cells, it was reasonable to hypothesize that similar deregulation is associated with development of resistance to chemotherapy. A report using used ovarian cancer cell lines Hey, Igrov-1, and SKOV-3 as a relative model for different levels of cisplatin-resistance, i.e., sensitive, intermediate-resistant and resistant, described the potential role of lipogenesis to this process [77]. Pre-treatment of cisplatin-resistant Hey-cis generated in vitro with FASN inhibitor cerulenin reversed platinum resistance, whereas no changes were observed in the parental cells. Exogenous addition of palmitic acid, one of the major products of FASN, rescued the re-sensitization to cisplatin caused by combined treatment with cerulenin and cisplatin, supporting the involvement of fatty acid synthesis in this phenomenon. In a more recent study, Papaevangelou et al. used xenograft models generated from cisplatin-resistant A2780-cis cells [78]. Intraperitoneal administration of the FASN inhibitor orlistat together with cisplatin significantly reduced tumor growth and tumor burden in these tumors. Interestingly, monotherapy with orlistat or with the combination decreased the abundance of hydrophilic metabolites involved in glutamine metabolism in addition to decreasing FASN activity and fatty acid production.

The role of fatty acid metabolism was also studied in other cancer types. For example, Wu et al. found that FASN mediated cellular responses to cisplatin treatment in breast cancer cells [41]. The underlying mechanism involved the upregulated transcription factor, specificity protein 1 (SP1) which was upregulated by FASN and, in turn, induced the expression of poly(ADP-ribose) polymerase 1. The later promoted recruitment of repair proteins Ku70 and DNA-dependent protein kinase at sites of double-strand breaks, initiating non-homologous end joining DNA repair machinery. These data link lipogenesis to the process of DNA damage response, important to determining sensitivity to platinum.

Furthermore, recent reports have suggested that drug-tolerant cells adopt a mesenchymal state and are dependent on lipid peroxidation mediated by the lipid hydroperoxidase GPX4 [79]. Targeting GPX4 induced the process of ferroptosis which selectively eliminated the “persister” cells responsible for treatment resistance in vitro and in vivo models. Interestingly, inhibition of the enzyme SCD1 in ovarian cancer cells induced both ferroptosis and apoptosis and combined inhibition of SCD1 with inducers of ferroptosis induced potent tumor inhibition in ovarian cancer models [80]. These examples strongly support the involvement of lipogenesis in response to chemotherapy and suggest that inhibitors for these pathways may be added to the armamentarium of cancer therapy in combination with chemotherapy.

## 4. Targeting Lipid Metabolism in Ovarian Cancer

Given the importance of lipid metabolism to tumor progression, in the past few years efforts have been channeled towards the development of small molecule inhibitors targeting different enzymes involved in this metabolic network. The compounds listed in Table 1 are under current investigation either in preclinical or clinical stages. More compounds, not discussed here, are undergoing structure-activity relationship development and optimization, as the field is evolving. Inhibitors of fatty acid biosynthesis include inhibitors for FASN, Acetyl CoA Carboxylase, SCD1, sterol regulatory element-binding protein 1 (SREBP1), CPT1, and ATP citrate lyase. Multiple FASN inhibitors are currently in clinical development, including GSK 2194069, IPI-9119, and TVB-2640. The latter is being studied for the treatment of non-alcoholic steatohepatitis and was assessed in a phase I clinical trial in patients with solid tumors, either alone or in combination with paclitaxel. TVB-2640 was reasonably well tolerated and most common adverse event were alopecia, palmar-plantar rash, and decreased appetite. Responses were observed in patients with ovarian cancer (5 of 12 patients), breast cancer (3 of 14 patients), and KRAS mutated lung cancer [81]. Based on these promising results, further development of this agent includes combinations with bevacizumab in glioblastoma and with paclitaxel and trastuzumab in breast cancer.

Acetyl-CoA carboxylase is indirectly targeted by metformin and directly blocked by specific inhibitors ND-646 and ND-654. The latter are being studied in preclinical models of lung cancer and hepatoma, while metformin has been studies extensively in combination with chemotherapy in clinical trials in breast, ovarian, and endometrial cancer or as a preventive cancer agent. Multiple inhibitors are also under development for SCD1 and SREBP1; however, none has yet entered the clinical arena. Inhibitors for ATP citrate lyase are being developed as cholesterol lowering drugs, with the most advanced being ETC-1002, a first in class compound, which completed phase III studies, demonstrating efficacy in blocking cholesterol synthesis [82]. Evaluation of this agent in cancer contexts has not yet been initiated.

CPT1 is the rate limiting enzyme controlling fatty acid oxidation, which is a major source of energy for several tumor types. The best studied CPT1 inhibitor is etomoxir which has been evaluated in preclinical models of prostate, breast, and bladder cancer and leukemia [83]. Etomoxir was found to synergize with glutaminase inhibitors in triple negative breast cancer cells [84]. The agent also inhibited proliferation of prostate cancer cells, especially under hypoxia [83], and blocked the proliferation of chronic lymphocytic leukemia cells resistant to ibrutinib [85]. Further exploration of inhibitors blocking fatty acid oxidation in various cancer contexts is warranted.

Interestingly, inhibition of lipid metabolism can also affect normal cells, particularly the immune system, including T cells, tumor-associated macrophages, regulatory T cells and myeloid derived stromal cells (MDSCs). An example is the fatty acid transport proteins which are upregulated in MDSCs, cells with immunosuppressive roles in tumors [86]. By reducing this cell population, inhibitors of FATP2, currently under preclinical development, could augment the effects of immunotherapy by selectively eliminating MDSCs. While at high doses, etomoxir was also shown to inhibit the survival of T regulatory and T memory cells as well as the polarization of macrophages, more recent work demonstrated that these effects were independent of CPT1 [87,88], refuting the previously proposed role of fatty acid oxidation in these processes. Future development of combinations of immunotherapy strategies with agents targeting lipid metabolism is a new direction beginning to be explored in cancer treatment.

## 5. Conclusions

In summary, emerging reports support the role of lipid metabolism in cancer stemness and metastasis. Several alterations in key molecules regulating pathways involving lipid uptake (CD36, FABP4), lipid synthesis (FASN) or desaturation (SCD1), or fatty acid oxidation (CTP1) have been described in ovarian cancer and linked to peritoneal dissemination, survival of stem cells and response to chemotherapy. Development of strategies targeting these key enzymes or lipid transporters, alone or in combination with other biologics or chemotherapy, is ongoing.

## Figures and Tables

**Table 1:** Inhibitors targeting different enzymes in the lipid metabolism network with their known IC_50_ and preclinical models and clinical studies.

Target	Compound Name	IC_50_	Preclinical Models or Clinical Trials	Refs
Acetyl-CoA carboxylase	Metformin	NA	Advanced pancreatic cancer, phase IB Prostate cancer, phase II Non-small-cell lung cancer, phase II Papillary renal cell carcinoma, phase I/II Colorectal cancer, phase II High-grade serous ovarian, or peritoneal cancer, phase II Advanced melanoma, phase I Head and Neck Squamous Cell Carcinoma, phase I/II Advanced stage ovarian cancer, phase II	[89,90,91,92,93,94,95,96]
	ND-646	3.5 nM	Non-small-cell lung cancer cells	[97]
	ND-654	3 nM	Liver cancer cells	[82]
Fatty acid synthase	GSK2194069	15nM	Non-small-cell lung cancer cells	[98]
	JNJ-54302833	28 nM	Ovarian and prostate cancer cells, lung cancer xenograft mice	[99]
	IPI-9119	0.3 nM	metastatic castration-resistant prostate cancer cells and xenograft mice	[100]
	TVB-2640	NA	Colon cancer, phase I HER2-positive advanced breast cancer, phase II Refractory high grade astrocytoma, phase II	[101,102,103]
Sterol regulatory element-binding protein 1	Fatostatin	11.2 μM	Prostate cancer cells, subcutaneous xenograft mice model	[104,105]
	Betulin	10s’ μM range	Different types of cancer cells Different types of primary tumor cells Different types of xenograft mouse models	[106]
	PF429242	24.5 μM	Pancreatic cancer cells	[107]
Stearoyl-CoA desaturase	MF-438	3–220 nM	Non-small cell lung cancer cells Melanoma cells	[108,109,110]
	MK-8245	1.066 μM	Liver cancer cells	[111]
	SAR707	39 nM	Liver cancer cells Obese Zucker diabetic fatty rats	[112]
Peroxisome proliferator-activated receptor α	TPST-1120	NA	Different types of advanced cancer, phase I	[113]
Carnitine palmitoyltransferase 1	Etomoxir	NA	Prostate cancer cells	[83]
ATP citrate lyase	ETC-1002	2–13 μM	Primary rat hepatocytes, obese female Zucker rat Hypercholesterolemia, phase III	[114,115]
	NDI-091143	7 nM	Precursors tested in liver cancer cells, high-fat diet fed mice. In vitro biochemical assay	[116,117]
